# Clinical impact of tissue sodium storage

**DOI:** 10.1007/s00467-019-04305-8

**Published:** 2019-07-30

**Authors:** Rik H. G. Olde Engberink, Viknesh Selvarajah, Liffert Vogt

**Affiliations:** 1 Location AMC, Department of Internal Medicine, Section Nephrology, Amsterdam Cardiovascular Sciences, Amsterdam UMC, University of Amsterdam, Amsterdam, The Netherlands; 2 Division of Experimental Medicine and Immunotherapeutics, University of Cambridge, Cambridge, UK

**Keywords:** Sodium, Skin, Glycocalyx, Endothelial surface layer, Nonosmotic, Glycosaminoglycan, Blood pressure

## Abstract

In recent times, the traditional nephrocentric, two-compartment model of body sodium has been challenged by long-term sodium balance studies and experimental work on the dermal interstitium and endothelial surface layer. In the new paradigm, sodium can be stored without commensurate water retention in the interstitium and endothelial surface layer, forming a dynamic third compartment for sodium. This has important implications for sodium homeostasis, osmoregulation and the hemodynamic response to salt intake. Sodium storage in the skin and endothelial surface layer may function as a buffer during periods of dietary depletion and excess, representing an extra-renal mechanism regulating body sodium and water. Interstitial sodium storage may also serve as a biomarker for sodium sensitivity and cardiovascular risk, as well as a target for hypertension treatment. Furthermore, sodium storage may explain the limitations of traditional techniques used to quantify sodium intake and determine infusion strategies for dysnatraemias. This review is aimed at outlining these new insights into sodium homeostasis, exploring their implications for clinical practice and potential areas for further research for paediatric and adult populations.

## Introduction

**Fig. 1:**
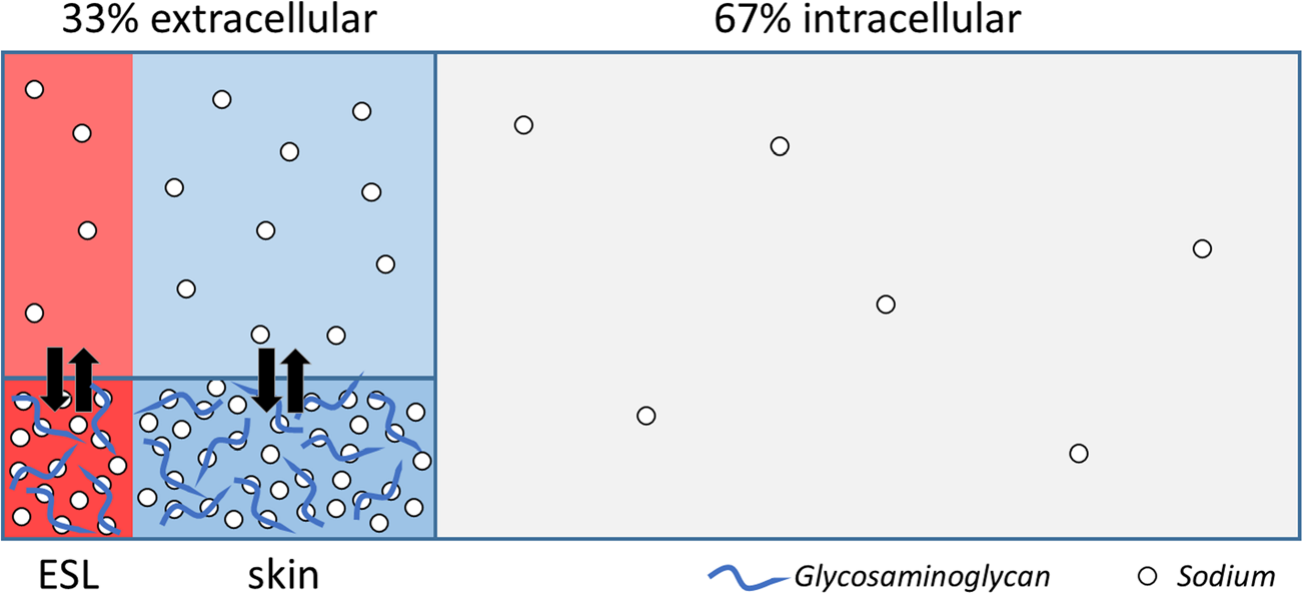
Sodium storage in a third compartment. The extracellular fluid compartment consists of an interstitial (blue) and intravascular (red) space that were considered to be osmotically equilibrated. However, significant higher sodium concentrations can be found in the skin and endothelial surface layer (ESL), comprising a third compartment that is not in osmotic equilibration with the other fluid compartments

In the last 15 years, sodium homeostasis has been completely revised going back to concepts that were first introduced in the early 1900s. The discovery of a third compartment in which sodium can accumulate without concurrent water retention is in sharp contrast with the two-compartment model that has been described in medical textbooks since the 1950s (Fig. 1). Although the notion of a third compartment is not new and has already been demonstrated a century ago, recent studies have shown that this third compartment has major consequences for daily clinical practice.

## Textbook sodium homeostasis

The current concepts of sodium homeostasis that are taught every day to medical students are based on the two-compartment model, which assumes that the total body water is divided over the intracellular (2/3rd) and extracellular compartment (1/3rd) with a similar osmolality. In the intracellular compartment, the main cation is potassium and sodium concentrations are low. The opposite is true for the extracellular compartment where sodium is the main cation and preserves effective circulating volume. According to the two-compartment concept, an increase in sodium intake or hypertonic NaCl infusion will add sodium to the extracellular compartment, increase extracellular osmolality and induce a water shift from the intracellular to the extracellular compartment to control plasma osmolality, which will only slightly increase. Following this increase in plasma osmolality, thirst sensation will lead to increased water intake, and the kidney will retain water in response to antidiuretic hormone release. As a result of a subsequent increase in total body water, plasma osmolality will return to baseline values at the expense of an expanded extracellular fluid volume and rise in blood pressure. This blood pressure increase, in turn, will induce pressure natriuresis and lower total body sodium content. These mechanisms have been first described by Borst and later on by Guyton who both have demonstrated that long-term control of arterial pressure is closely related to body fluid homeostasis [1, 2].

As outlined above, adopting the traditional two-compartment model implies that the kidney is crucial for regulation of both water and sodium balance and should be perfectly able to match sodium and water excretion with the perceived intake resulting in a ‘zero balance’ during steady-state water and sodium intake. Disruption of this balance will lead to problems with volume or osmoregulation. For example, sodium sensitivity of blood pressure is believed to be attributed to impaired renal sodium excretion.

## New insights into sodium homeostasis

Long-term sodium balance studies, however, have demonstrated that sodium and water homeostasis cannot be explained by the two-compartment model and is more complicated [3–5]. These studies, which carefully measured sodium intake and excretion during 200 consecutive days in an enclosed habitat, demonstrated that 24-h sodium excretion can differ up to 80 mmol from 24-h sodium intake during stable sodium intake, thereby inducing large fluctuations in total body sodium content up to 1000s of mmols over weeks. Surprisingly, a matching increase in total body water (1 L for every 140 mmol sodium) and extracellular volume that would be expected according to the two-compartment model was not observed. Also, changes in total body sodium content were not related to blood pressure.

Experimental studies provided an explanation for these unexpected findings. In the skin interstitium, sodium accumulation was associated with increased content and sulfation of negatively charged glycosaminoglycans (GAGs) [6]. Via binding to these GAGs, sodium can be osmotically inactivated and does not induce concurrent water retention (i.e. nonosmotic sodium storage). As the skin is a large organ and skin sodium concentrations up to 180–190 mmol/L were found, a significant amount of sodium can be stored without effects on extracellular volume, body weight or blood pressure [6]. Monocytes, which seem to be attracted by high interstitial sodium concentrations, play a crucial regulating role in skin sodium homeostasis. Once in the skin, macrophages modulate vascular endothelial growth factor-C (VEGF-C)–mediated hyperplasia of lymph vessels, which is considered to be the principal process of mobilization of excessive sodium from the skin [7]. Interestingly, an increasing body of evidence suggests that a significant amount of the skin sodium excess is not osmotically inactivated by GAGs but is actively concentrated in the skin by a kidney-like countercurrent system and serves as a hypertonic barrier that prevents skin water loss [8–11]. This theory may also explain the large fluctuations in total body chloride, as chloride can be concentrated in the skin too [10].

In addition to the skin, the endothelial surface layer (ESL) is likely to be involved in nonosmotic sodium storage [12]. The ESL is a dynamic layer consisting of GAGs, proteoglycans and adsorbed plasma proteins covering the inner surface of the endothelium. In contrast to the skin interstitium to which sodium has to be transported, the ESL may provide capacity for *instant intravascular* sodium storage. As this vascular system for sodium storage has a volume of 1.5 L in healthy subjects, significant amounts of sodium can be inactivated right after sodium has entered the circulation [13]. The observation that diseases that are characterized by ESL damage, such as diabetes mellitus and chronic kidney disease, are often associated with volume overload may indicate that sodium inactivation by the ESL GAGs contributes to preserving normal volume regulation [13–15].

The exact role of sodium storage in the skin and ESL and the interaction between both compartments is unknown. We recently hypothesised that skin sodium accumulation, which may seem beneficial at first sight, is harmful and is likely to represent significant sodium excess and impairs vascular function [16]. This is supported by the observation that an increased skin sodium content is observed in patients with fluid overload such as hyperaldosteronism, acute kidney injury, dialysis and heart failure patients [17–20]. Moreover, high skin sodium content is strongly associated with left ventricular hypertrophy [21]. Conversely, nonosmotic sodium storage in the ESL seems to be crucial to reduce the negative effects of sodium excess such as an increase in extracellular volume and blood pressure [16]. Also, the ESL has an important barrier function and may thus prevent skin sodium accumulation [16].

In clinical practice, physicians need to deal with disturbances of osmoregulation and volume regulation on a daily basis, particularly in subjects with hypertension, kidney disease, or heart failure or critically ill patients. The novel insights into sodium homeostasis may significantly impact daily clinical practice and could provide an explanation for so far inexplicable findings, but may also provide new diagnostic or therapeutic options. We will review the prevalence and importance of (nonosmotic) sodium storage in common diseases and the effect of frequently used therapies and discuss the clinical consequences for dysnatremias, hypertension and sodium intake estimation using urine samples.

## Interstitial sodium storage and osmoregulation

The presence of an additional compartment in which sodium can be (temporarily) stored complicates diagnostics and treatments that are based on the two-compartment model. In 1958, Edelman et al. described the relation between serum osmolality and the ratio of total body exchangeable cation content and total body water [22]. On first sight, these data support the concept that serum osmolality is only influenced by water, sodium and potassium. However, an important limitation of the Edelman study is that measurements were performed in steady-state conditions, meaning that subjects with hyponatremia, normonatremia and hypernatremia were compared, whereas infusion of hypo- or hypertonic solution *within* a subject may result in different data given the plasticity of the newly discovered third compartment. In the latter case, sodium may be mobilized from interstitial sodium storages in response to hypotonic stimuli, whereas excess sodium may be stored after hypertonic stimuli [23, 24]. As a consequence, the individual patient may not move along the regression line of the Edelman equation following infusion therapy as the relation between serum osmolality, total body cation and total body water content may be significantly altered due to the temporary storage and release of sodium.

The Edelman equation is the basis of formulas that are currently used in daily clinical practice to estimate the effect of sodium or water infusion in case of hypo- or hypernatremia, such as the Adrogue-Madias, Nguyen-Kurtz and Barsoum-Levine formulas [25–27]. However, multiple studies have demonstrated that these formulas are not able to accurately estimate changes in plasma sodium concentration. In healthy subjects, hypertonic saline infusion induced changes in plasma sodium concentration that were on average 2.2 mmol/L different from the expected values within 2 h after infusion [28]. Also, the observed changes in plasma sodium concentration were not in line with the urine cation excretion that was expected according to these changes. Despite the fact that 108 mmol of sodium was cleared from the total body water during a 4-h period, only 51 mmol of sodium was retrieved in the urine. These data indicate that healthy subjects have a significant capacity for interstitial sodium storage that can be utilized in situations of sodium excess such as sodium infusion or high sodium diet. Recently, we demonstrated that interstitial sodium storage is also involved in prevention of acute hypotonicity. After oral water loading in healthy subjects, the observed decrease in plasma sodium concentrations was 60% less than expected according to the traditional two-compartment model, indicating recruitment of sodium from interstitial or ESL stores [29].

Data from clinical studies demonstrate that even larger discrepancies are seen in daily clinical practice. Both in hypo- and hypernatremic patients the observed plasma sodium concentrations were > 2 mmol/L higher than expected within 24 h after initiating treatment [30]. In a subgroup of 15 volume-depleted hyponatremic subjects, the average inconsistency was even 5.6 mmol/L. A subsequent study showed that only 50% of the variability of the observed plasma sodium concentration could be explained by the estimated values [31]. The average discrepancy between estimated and observed plasma sodium concentrations in this study was 3.4–4.5 mmol/L for hyponatremic patients and 5.0–6.7 mmol/L for hypernatremic patients depending on the formula that was used. The crucial role of the third compartment in the pathophysiology of dysnatremias is confirmed by a case report of a hypernatremic patient in which skin and muscle sodium content were significantly increased during hypernatremia but normalized after correction of hypernatremia [32]. The inaccuracy of the current formulas in the clinic therefore seems a logical consequence of the fact that the Edelman equation does not take into account storage and release of sodium from a dynamic third compartment. As both overcorrection and undertreatment of dysnatremias are harmful and may even be lethal, it is crucial that the effects of treatment on plasma sodium concentration are frequently monitored to timely recognize potentially unpredictable changes.

To improve patient care, further research needs to identify factors that affect osmoregulation in addition to total body water, plasma sodium and potassium concentration, and sodium and potassium excretion. This could include patient characteristics that have been demonstrated to affect interstitial sodium storage such as age, gender, sodium intake, blood pressure, diabetes mellitus, infection and inflammation [17, 19, 33–39]. These additional variables may help to estimate the treatment effects of hypo- and hypertonic saline infusion more accurately.

## Tissue sodium storage and volume regulation

The new insights into sodium homeostasis challenge the (patho)physiology underlying blood pressure regulation and hypertension, in particular the effect of sodium intake on blood pressure, also known as sodium sensitivity. Decades of research have not resolved the phenomenon of sodium sensitivity. This may be explained by the fact that most research was focussed on the kidney as an impaired renal capacity for sodium excretion was thought to be responsible for sodium sensitivity. The findings that total body osmotically active sodium content is not only regulated by the kidney, and that total body sodium content is not necessarily related to blood pressure, provide new insights into potential mechanisms responsible for sodium sensitivity. Recently, interests have shifted to the skin interstitium and ESL as potential modulators of sodium sensitivity [12, 40].

Laffer et al. have put forward the vasodysfunction theory that links interstitial sodium accumulation with vasodysfunction and ultimately sodium sensitivity [40]. In this study, sodium loading and depletion were tested in sodium-sensitive and sodium-resistant individuals. In contrast to sodium-resistant individuals who were able to lower peripheral resistance in response to sodium loading thereby preserving normal blood pressure, sodium-sensitive individuals could not modulate peripheral resistance resulting in a blood pressure increase. Even more interesting is that sodium loading in the sodium-resistant subjects did not affect body weight whereas an iso-osmolar retention of water (1 L per 140 mmol sodium) was observed in sodium-sensitive individuals. These findings suggest that sodium-resistant subjects have a residual capacity for neutralization of a sodium load without concurrent water retention while in sodium-sensitive subjects interstitial sodium storage is fully saturated. This hypothesis is supported by data from multiple ^23^Na-MRI studies that have demonstrated interstitial sodium accumulation in subjects that are known to be sodium sensitive such as the elderly and hypertensive, diabetic, heart failure and dialysis patients [17–19]. Besides the iso-osmolar increase in extracellular volume in sodium-sensitive subjects, the high interstitial sodium concentration itself may contribute to a sodium-induced increase in blood pressure. Previous studies have demonstrated detrimental effects of hypersalinity on endothelial function that may subsequently impact total peripheral resistance and blood pressure [41, 42]. Altogether, these data indicate that changes in peripheral resistance do not merely seem a consequence of long-term autoregulation to tissue hyperperfusion, as was believed according to the two-compartment model theory, but may be directly responsible for blood pressure changes in sodium-sensitive individuals.

Although interstitial storage of sodium is a new, and for many clinicians unknown concept, it is likely that most clinicians have actively altered interstitial sodium content in their patients last month. Data from ^23^Na-MRI studies show that everyday treatments such as diuretics, sodium glucose cotransporter 2 (SGLT-2) inhibition and dialysis significantly impact interstitial sodium content [17, 18, 43]. These therapies may be of particular interest in sodium-sensitive hypertension as these subjects are characterized by interstitial sodium accumulation. Until recently, it was unknown whether interstitial sodium accumulation contributes to the cardiovascular risk. Yet, a recent trial demonstrated an association between skin sodium content and an intermediate endpoint, left ventricular hypertrophy. In chronic kidney disease patients, interstitial sodium content was strongly correlated with left ventricular mass, independent of blood pressure or total body overhydration [21]. The observed correlation was stronger than the correlation between total body overhydration and left ventricular mass. Although we need to wait for data from long-term studies investigating the potential cardiovascular risk that is associated with interstitial sodium accumulation, these data are interesting, in particular because interventions are possible with commonly used therapies.

## Nonosmotic sodium storage and hypertension treatment

The novel insights into sodium homeostasis may also lead to new treatment options for hypertension. In this respect, the ESL is of interest as previous studies have demonstrated that the ESL volume in diabetic and chronic kidney disease patients is significantly reduced [13, 15]. Many studies have attempted to restore the ESL and thereby its important barrier functions by oral supplementation of GAGs. In type 2 diabetic patients, a highly purified mixture of GAGs named sulodexide has been shown to be able to restore the damaged ESL [44]. For that reason, many clinical studies have investigated sulodexide, most of them focussing on the potential anti-albuminuric effects that were expected after restoration of the glomerular ESL in diabetic patients. Although small studies were promising, large randomized, placebo-controlled trials were not able to confirm the anti-albuminuric effects of sulodexide [45, 46].

Considering the ESL restoring capacity of sulodexide, it increases the intravascular capacity for nonosmotic sodium storage and may thereby decrease osmotically active sodium and potentially blood pressure. To investigate this hypothesis, we have meta-analysed studies that had investigated sulodexide for different medical conditions but also measured blood pressure [47]. In 8 placebo-controlled trials, of which 7 were double blinded, including 3019 subjects, we demonstrated that sulodexide lowered blood pressure significantly. This effect was observed despite the fact that the majority of patients were already being treated with maximally tolerated renin-angiotensin system inhibition and the majority had a normal blood pressure at baseline (i.e. < 140/90 mmHg). In subjects with actual hypertension, the decrease in systolic (10 mmHg) and diastolic (5 mmHg) blood pressure was similar to regularly prescribed antihypertensive therapy [48]. In a subsequent study investigating individual patient data of the 2 largest studies included in the meta-analysis, we demonstrated that the magnitude of albuminuria at baseline was an important modifier of the blood pressure response after sulodexide [49]. As previous studies have shown that the ESL thickness decreases with increasing amounts of albuminuria, these data suggest that the blood pressure reducing capacity of sulodexide can be attributed to the ESL restoring properties [13, 50]. Considering this new working mechanism that differs from regular antihypertensive drugs and may decrease sodium sensitivity, future research should point out whether sulodexide may be of added value to the antihypertensive drugs that are currently available.

## Estimation of sodium intake

**Fig. 2:**
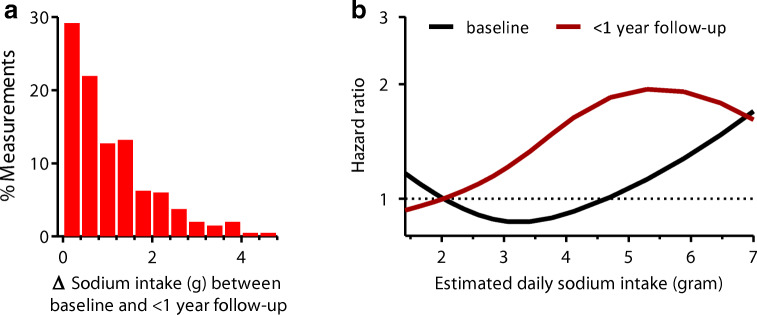
Consequences of estimating sodium intake with a single measurement. **a** Differences in estimated sodium intake when estimated at baseline or within 1 year after follow-up. **b** Differences in the associated risk for the composite of cardiovascular events and death when using baseline or 1-year follow-up estimates of sodium intake. Adapted from [52]

A single collection of 24-h urine is regarded as the gold standard for estimation of sodium intake and is widely used in clinical practice and cohort studies. This method is based on the assumption that 24-h sodium intake equals 24-h sodium excretion during stable sodium intake. However, long-term sodium balance studies have shown that this assumption is incorrect as 24-h sodium excretion may be up to 80 mmol different from the actual intake *during fixed intake* because of infradian rhythms in total body sodium content induced by aldosterone and cortisol [3]. On top of these fluctuations, day-to-day variations in sodium intake, medication effects and collection errors complicate the use of a single 24-h urine measurement for estimation of sodium intake. A subsequent analysis that investigated multiple 24-h urine collections during fixed sodium intake demonstrated that 7 consecutive 24-h urine collections were needed for estimation of steady-state sodium intake once [51]. The need for multiple measurements is further emphasized by a cohort study investigating outpatients who collected multiple 24-h urine samples during a 17-year follow-up [52]. Baseline and follow-up estimates of sodium intake were > 34 mmol different in half of the subjects (Fig. 2). This inconsistency was present both when estimating follow-up sodium intake estimates within 1 year after baseline and when analysing 15-year average estimates. In daily practice, clinicians should therefore not rely on a single 24-h urine collection for estimating sodium intake and making therapeutic decisions but should base treatment and dietary advice on multiple measurements.

These findings also complicate the use of single urine collections for research purposes. Although a single urine collection can be used for estimation of population sodium intake, individual estimates based on a single urine collection are inaccurate and significantly impact research outcomes. This is illustrated by the abovementioned study that investigated the association between sodium intake and cardiovascular outcome, both when estimating sodium intake with a single baseline collection and when estimating sodium intake with multiple 24-h collections during long-term follow-up [52]. Major differences in the observed associations were seen. Whereas the highest tertile of estimated sodium intake was not associated with cardiovascular disease or mortality when sodium intake was assessed at baseline (relative risk 1.09, 95% confidence interval 0.61–1.95), a significant association was observed when sodium intake was estimated within 1 year after baseline (RR 1.80, 95% CI 1.03–3.13). The major influence of the method used for estimation of sodium intake may, at least in part, explain the inconsistent findings of cohort studies that have investigated the relation between sodium intake and cardiovascular outcome, but have used varying methods for estimation of sodium intake. Given the inaccurateness of a single urine collection, future studies should use multiple urine collections when assessing individual sodium intake.

## Potential implications of tissue sodium storage in paediatrics

These novel insights into sodium homeostasis may have potential implications for clinical practice and future research in paediatrics. Studies looking at interstitial sodium storage in children are lacking with most research done in adults. A previous meticulous dietary salt modulation study in healthy girls aged 11–15 of black and white race showed that body sodium retention occurred without weight gain or a rise in blood pressure, raising the possibility that interstitial sodium storage functions in adolescence [53]. In this study, greater sodium retention was observed with black race, which could indicate the existence of racial differences in interstitial sodium storage before adulthood. It is unclear how sodium storage first develops in human interstitial and vascular systems or its function in sodium homeostasis in neonates, who are prone to renal sodium losses from tubular immaturity [54]. It may be relevant to consider interstitial sodium storage in salt wasting nephropathies such as Bartter syndrome, particularly with regard to whether the interstitium acts as a buffer for renal sodium losses.

The relevance of the ESL in children has been suggested by a previous study which showed a significant reduction in endothelial glycocalyx thickness by 36% in children with type 1 diabetes compared with controls [55]. This reduction appeared to precede the onset of microalbuminuria and hypertension, raising the possibility that restoring the ESL with agents such as  sulodexide may be a therapeutic option to prevent these problems developing later in life.

The association between sodium intake and blood pressure in children has been previously reviewed [56]. As in adults, dietary sodium consumption in children has been observed to be above recommended levels in developed countries, with sodium intake being shown to be positively associated with blood pressure [57–59]. It is unknown whether interstitial sodium storage modulates sodium sensitivity in childhood and adolescence, or determines hypertension in later life. Studies in adults have shown that interstitial sodium accumulation in the skin and muscle increases with age and positively correlates with blood pressure [19, 21]. Longitudinal studies evaluating interstitial sodium in children and adolescents using methods such as ^23^Na-MRI could reveal the direction of causality in the relationship between interstitial sodium and blood pressure, showing if interstitial sodium accumulation starts early in life and precedes, or is implicated in the development of hypertension. This may further strengthen the need for preventative strategies such as dietary sodium reduction in children to prevent the onset of hypertension in adulthood. Recent studies have shown a higher prevalence of sodium sensitivity in women with possible differences in interstitial sodium accumulation and storage [34, 35, 60]. It may be relevant and informative to explore these sex differences in children, before the onset of puberty and major influence of sex hormones or size differences.

## Conclusion

Interstitial and ESL sodium storage is a clinically relevant concept that complicates treatments and diagnostics based on the classical two-compartment model but may also provide new possibilities for treatment of common diseases such as hypertension. The current understanding of the impact of (nonosmotic) sodium storage on daily clinical practice is likely to be only a fraction of the impact it actually has.
